# Drug Resistance: The Role of Exosomal miRNA in the Microenvironment of Hematopoietic Tumors

**DOI:** 10.3390/molecules28010116

**Published:** 2022-12-23

**Authors:** Mariaconcetta Cariello, Angela Squilla, Martina Piacente, Giorgia Venutolo, Alessio Fasano

**Affiliations:** 1 European Biomedical Research Institute of Salerno (EBRIS), Via S. de Renzi, 84125 Salerno, Italy; 2 Mucosal Immunology and Biology Research Center, Massachusetts General Hospital for Children, Boston, MA 02114, USA

**Keywords:** drug resistance, tumor microenvironment, hypoxia, exosomes, miRNAs

## Abstract

Extracellular vesicles (EVs), including exosomes, have an important role thanks to their ability to communicate and exchange information between tumor cells and the tumor microenvironment (TME), and have also been associated with communicating anti-cancer drug resistance (DR). The increase in proliferation of cancer cells alters oxygen levels, which causes hypoxia and results in a release of exosomes by the cancer cells. In this review, the results of studies examining the role of exosomal miRNA in DR, and their mechanism, are discussed in detail in hematological tumors: leukemia, lymphoma, and multiple myeloma. In conclusion, we underline the exosome’s function as a possible drug delivery vehicle by understanding its cargo. Engineered exosomes can be used to be more specific for personalized therapy.

## 1. Introduction

Cancer is the principal cause of mortality worldwide, accounting for nearly 10 million deaths in 2020, or nearly one in six deaths [1], with drug resistance (DR) being one of the main contributors to its increase [2]. Cancer cells are initially sensitive to therapy, but progressively develop a DR phenotype through different mechanisms. Furthermore, some neoplasms even develop a phenotype resistant to multiple antitumor drugs, with distinct characteristics and structures. This phenomenon, known as multi-drug resistance or multiple-drug resistance (MDR), is a major challenge in the cancer treatment of many types of hematological neoplasms [3,4]. It can result from genetic or phenotypic changes in cancer cells [5], or from remodeling within the tumor microenvironment (TME) [6,7]. The TME is an environment which constitutes a complex system made up of different types of cells, signal molecules, and mediators. Communication between cancerous and surrounding cells involves various mechanisms that lead to tumor growth and DR [8,9,10]. Cells in the TME exchange information through a variety of signaling networks such as cell–cell junctions, secreted factors, and extracellular vesicles such as exosomes [11,12,13]. As demonstrated by recent studies, exosomes are a type of nano-sized vesicle that play an important role in the communication between tumor cells and the TME. Their importance in hematology/oncology is attributed to their possible use as biomarkers of disease progression and DR [14,15]. They have the inherent potential to shuttle different biomolecules such as lipids, proteins, and nucleic acids such as DNA, messenger RNA (mRNA), and micro-RNA (miRNA) in between neoplastic cells themselves, or to different compartments of the bone marrow (BM) where they create a premetastatic niche that supports cell proliferation and DR [16,17,18,19]. As exosomal miRNA reflects the miRNA signature of the parental tumor [20], it can induce DR by transporting specific genetic traits from a DR-cell to a sensitive one [8], thus promoting the progression of cancer and therapy resistance [21,22,23]. In this review, we illustrate the relationship between tumor DR and exosomes, examine current knowledge on the involvement of miRNA in DR and, ultimately, discuss the potential applications of exosomes as therapeutic targets in hematological neoplasms.

## 2. Hematological Tumors: Types and Features

In various hematological tumors, exosomes can create a permissive microenvironment in the BM with enhanced angiogenesis, increased immune suppression, and increased osteolysis. In this way, the proliferation of tumor cells is allowed and, therefore, so is the progression of the neoplasm [24]. In this review, we will focus specifically on the following hematological malignancies and the mechanisms of resistance to therapy related to them.

### 2.1. Leukemia

Leukemia is a type of hematological malignancy caused due to the excessive production of abnormal white blood cells. In 2017, leukemia represented 30.4 percent of blood cancers, and 2.9 percent of other cancers in the USA. The term leukemia is used to reference a broad array of hematopoietic malignancies currently subcategorized into acute myeloid leukemia (AML), chronic myeloid leukemia (CML), acute lymphoblastic leukemia (ALL), and chronic lymphocytic leukemia (CLL) according to their morphology, immunophenotype, cytogenetic and molecular abnormalities, and clinical features which help to dictate the approach to treatment [25].

#### 2.1.1. Acute Myeloid Leukemia

Acute myeloid leukemia (AML) is one of the most common malignant blood cancers which results in a high mortality rate [26]. It is characterized by incomplete maturation of myeloid cells that occurs due to the clonal over-proliferation of an aberrant stem cell engaged at the level of colony forming unit (CFU) or later stages of differentiation. This situation leads to the accumulation and clonal expansion of non-functional myeloblasts, known as blasts, that accumulate in the BM and blood [27]. AML induces multipotent mesenchymal stem/stromal cells (MSC) to differentiate into adipocytes rather than osteoblasts, with a metabolic shift from glycolysis to oxidative phosphorylation (OXPHOS) during energy production. The enhanced adipogenesis of MSC represents one of the mechanisms of increased engraftment of leukemic cells; in fact, the adipocytes of the BM have been reported to support the survival and proliferation of AML blasts. The progression of leukemia is guaranteed by the exosomes produced by the AML cells that facilitate the engraftment of AML and allow the communication of leukemia cells with their microenvironment, as well as inducing oncogenic factors such as c-Myc. [25,28]. Furthermore, the release of exosomes leads to the production of immune-inhibiting cytokines, thus ensuring the growth and survival of cancer cells. The presence of exosomes in TME, therefore, indicates an unfavorable prognostic factor which results in disease progression [26].

#### 2.1.2. Chronic Myeloid Leukemia

Chronic myeloid leukemia (CML) accounts for 10–15% of all leukemias. It is a myeloproliferative disease characterized by the presence of a molecular rearrangement, known as the Philadelphia chromosome (Ph), that generates a BCR/ABL fusion gene and the production of the BCR/ABL fusion protein with tyrosine kinase activity. Several studies have indicated that BCR/ABL activity contributes to several characteristics observed in CML, such as inhibition of apoptosis, disorganization of the cytoskeleton, decrease in cell adhesion, and differentiation, etc. [29]. Despite the clinical efficacy achieved by therapies with tyrosine kinase inhibitors (TKIs), the disease often progresses to the accelerated or blast and acute phase, following the development of DR mechanisms that lead to treatment failure [30].

#### 2.1.3. Acute Lymphoblastic Leukemia

Acute lymphoblastic leukemia (ALL) is one of the most frequent hematological diseases in children. It is very aggressive and occurs due to an accumulation of lymphoid progenitor cells in various tissues, particularly in the BM. Therapeutic improvements in recent years have increased survival rates in pediatric ALL. However, in adults there is a recurrence rate of more than 50% and an overall survival rate of 20–40%. Resistance to chemotherapy is a major obstacle for the treatment of relapsing ALL, and there is, therefore, an urgent need to develop new therapeutic modalities [31,32].

#### 2.1.4. Chronic Lymphocytic Leukemia

Chronic lymphocytic leukemia (CLL) is a B-cell lymphoproliferative disease characterized by the accumulation of mature B cells, showing a particular phenotype in the peripheral blood, BM, lymph nodes, and spleen that causes lymphocytosis. This buildup ensures easy diagnosis from peripheral blood. It is a disease that mainly affects the elderly with a 5-year survival rate of 81.7% [33].

### 2.2. Lymphoma

Malignant lymphoma is the most common hematological neoplasm and has heterogeneous phenotypes characterized by a few cells surrounded by immune cells, fibroblasts, stromal cells, and endothelial cells [34]. Lymphoma TME contributes to tumorigenesis, tumor cell survival, and drug resistance [35]. In relation to the presence and absence of the Reed Sternberg cell (RS), lymphomas are divided into Hodgkin’s lymphoma (HL) and non-Hodgkin’s lymphoma (NHL), respectively. HL typically presents Hodgkin (mononuclear) and Reed-Sternberg (multinucleated) cells. Reed-Sternberg cells are binucleated with a diameter greater than 50 µm but, as they constitute 1% of tumor tissue, it is difficult to study them. They survive, proliferate, and are able to generate a very aggressive and potentially lethal malignant tumor; they are protected from the cytotoxic effects of anticancer drugs thanks to the presence of soluble factors and the protection of the surrounding microenvironment, thus making this neoplasm incurable for about half of the patients [34,35,36]. Hodgkin and Reed-Sternberg cells can secrete cytokines/chemokines and angiogenic factors capable of recruiting adjacent cells and communicating with them through the release of exosomes. NHL is a lymphoid malignancy with multiple subtypes linked to differences in age, biology, and tumor survival. Non-Hodgkin’s lymphomas make up about 90% of cases and include many subtypes [34]. This kind of lymphoma includes Burkitt lymphoma, diffuse large B-cell lymphoma, lymphoblastic lymphoma, and anaplastic large cell lymphoma, that generally occur in children younger than 16 years; follicular lymphoma and marginal zone B-cell lymphomas are less common, but more frequent with increasing age. Some subtypes are not curable due to widespread DR; therefore, the inclusion of new agents in the standard treatment combinations is necessary to overcome the failure of current therapy [37,38].

### 2.3. Multiple Myeloma

Multiple myeloma (MM) is the second most common hematological malignancy in the United States and accounts for over 10% of all hematologic tumors [39]. It is characterized by the uncontrolled growth of monoclonal neoplastic plasma cells that infiltrate and accumulate in the BM. The pathophysiology of MM depends both on intracellular factors derived from genetic, epigenetic, and chromosomal alterations and on extracellular factors that allow for dynamic interactions between MM cells, and the BM microenvironment (BMME). The interaction between myeloma cells and BMME contributes to the progression of MM, the development of osteolysis and DR. Osteolysis is one of the main hallmarks of MM and derives from an interrupted homeostasis in bone formation and resorption [24]. Recent studies indicate that MM cells influence BM cells to model the BMME by promoting the growth and survival of MM cells [40]. In most MM patients, a premalignant state called Monoclonal Gammopathy of Indeterminate Significance (MGUS) is found, which precedes MM. Melanoma is one of the most chemo-resistant malignancies [41]. Conventional therapies, such as hematopoietic stem cell transplantation, result in a low complete remission. New therapies emerging from the development of new drugs, such as the proteasome inhibitor (PI) (bortezomib, carfilzomib) and immunomodulatory drugs (thalidomide, lenalidomide), can affect the BMME of MM and significantly improve the survival of MM patients [42,43]. However, MM remains an incurable malignancy with a 5-year survival rate of approximately 40%. [44,45]. Therapeutic resistance occurs in the majority of MM patients, and one of the key mechanisms of resistance to anticancer drugs is mediated by the TME, mainly by the release of exosomes by the many adipocytes residing in the BMME which inhibit the apoptosis of MM cells induced by chemotherapy. In particular, the bioactive components of exosomes released by adipocytes include lipids, proteins, miRNAs, mRNAs, long non-coding RNAs (lncRNAs), and DNA that influence the activities of target cells. Exosomal lncRNAs confer PI resistance to MM cells through structural changes in the proteasomal enzyme complex [46]. Furthermore, MM cells activate histone methylation in the promoter of adipokine genes in adipocytes. These reprogrammed adipocytes secrete adipokines and cytokines through their exosomes, promoting tumor progression and growth [39,47]. Many studies have shown that exosomes play an important role in BMME and suggest them as a new therapeutic target for anti-myeloma therapy. It has been found that in vivo blockade of exosome secretion, combined with bortezomib (Btz), led to a reduction in osteolysis and a significant antitumor effect [24].

## 3. Drug Resistance

Multi-Drug Resistance (MDR) is one of the most important barriers in medical oncology and, although impactful progress has been made to combat DR, there is still a long way to go to resolve the problem of resistance to drugs [48,49]. DR is always accompanied by the dysfunction of pharmacokinetic factors, which are drug absorption, distribution, metabolism, and elimination (ADME) [49]. Cancer cells may be naturally resistant to a certain drug or acquire resistance to that drug following treatment [6]. There are two types of resistance to anticancer drugs: primary or intrinsic resistance, meaning that a cancer cell is insensitive to that drug, and resistance acquired as a result of genetic and phenotypic changes in cancer cells as a result of a variety of adaptive responses, including modulation and activation of alternative signaling pathways [10,20]. The precise mechanisms of DR in cancer are complex and multifactorial and include genetic and epigenetic alterations that reduce or nullify the efficacy of the anticancer drug in cancer cells. The currently known mechanisms of MDR include alteration in DNA repair capacity [49], reduced susceptibility to apoptosis, altered expression of drug targets, MDR-protein overexpression, and increased cellular drug export. Indeed, excessive drug efflux, for example, is a classic DR mechanism [5,50]. Recently, studies have demonstrated that exosomes released in the TME play an essential role in tumor growth [5,18,51], metastasis, and resistance to anticancer drugs thanks to their ability to communicate between cancer cells and the TME [52,53]. Communication between the tumor and the TME, selective pressures caused by stressful conditions, acid TME, and exosome-mediated trait transfer are all factors related to DR mechanisms [4]. Exosomes are multi-signal messengers that participate in intercellular communication, increased drug export, and the transport of specific molecules that confer resistance to the target cell [11,49,53]. The rapid development of DR in cancer cells is one of the biggest obstacles of cancer treatment. At present, combination strategies for DR exist but the success of anticancer treatments depends on an in-depth knowledge of all the processes involved, and of the complex interactions between the components of the TME [48,54].

Hematological tumors recur in most patients due to the rapid acquisition of DR by tumor cells which, at the BM level, create an optimal microenvironment capable of playing an important role in anticancer DR, proliferation, and metastatic behavior of malignant tumors. Several mechanisms of resistance are largely mediated by miRNA contained in the exosomes of cancer cells. These mechanisms include downregulation of drug targets (see Section 3.1); cell cycle regulation, apoptosis, and DNA repair (see Section 3.2); reduction in the concentration of anticancer drugs (see Section 3.3); modulation of the TME (see Section 3.4). All these mechanisms can be grouped into three categories: (i) insufficiency of pharmacokinetic properties, (ii) intrinsic factors of tumor cells and iii) external conditions of tumor cells in TME [5,50].

### 3.1. Downregulation of Drug Targets

Downregulation of drug targets consequently makes the drug less active. The use of glucocorticoids is very frequent in anticancer therapies of hematological neoplasms. Their mechanism of action begins when they bind to the glucocorticoid receptor (GR), with consequent upregulation of pro-apoptotic genes that induce apoptosis of cancer cells. Some miRNAs are able to influence the expression of the GR gene, causing its deficiency, and therefore a lower action of glucocorticoids on the tumor [4,15]. In the presence of exosomes released from cancer-associated fibroblasts (CAFs), lymphoma cells have been shown to exhibit resistance to gemcitabine and cytarabine following downregulation of their transporter ENT2. In fact, some miRNAs contained in exosomes released by CAFs (such as miRNA-4717-5p) reduce ENT2 expression on tumor cells, causing chemoresistance [35].

### 3.2. Cell Cycle Regulation, Apoptosis, and DNA Repair

Exosomes can induce resistance by influencing cell cycle regulation, apoptosis, and DNA repair of cancer cells [55]. Exosomes are able to promote resistance to therapy-sensitive tumor cells by transmitting miRNAs that alter cell cycle control and induce anti-apoptotic programs. Furthermore, exosomes can trigger DNA repair. Indeed, in absence of immediate DNA repair, the accumulation of DNA damage in cancer cells following anticancer therapy results in a greater sensitivity to specific DNA-acting drugs [4,49]. Moreover, AML cell-derived exosomes contain a high concentration of miR-10b which suppresses hematopoietic stem cell differentiation and increases the proliferative ability of immature myeloid cells [35].

### 3.3. Reduction of the Concentration of Anticancer Drugs

The concentration of anti-cancer drugs can be reduced by decreasing their permeability across the cell membrane, or by increasing their active efflux [6]. Greater drug efflux is associated with increased expression of ATP-binding cassette (ABC) transporters. Several studies have shown that some miRNAs present in cancer cell-derived exosomes are able to increase the transcription of the P-glycoprotein (P-gp) gene of sensitive cells, inducing resistance in the latter through an increase in drug efflux. Furthermore, some miRNAs can also modulate drug efflux and thus DR, indirectly, by targeting proteins associated with drug transporters [4,6]. Resistance mechanisms in ALL involve miR-324-3p and miR-508-5p which regulate ABCA3 gene expression. This gene encodes the ABC transporter, resulting in drug efflux from the tumor cells of pediatric ALL patients [32].

### 3.4. Modulation of the Tumor Microenvironment

TME-mediated DR results from the combination of soluble factors produced by the interaction of tumor cells with TME, and by direct physical contact between cells, as well as by acidification of the microenvironment itself. These soluble factors can act on different cellular targets that regulate multiple processes such as cell growth and apoptosis, engraftment cancer, and angiogenesis, thus creating a permissive niche. As a result, the interplay of TME-soluble factors activates different pathways that lead to the protection of cancer cells and DR. Interleukin-6 (IL-6), for example, triggers the activation of the Ras/Raf/MEK/MAPK, JAK/STAT3 and Phosphatidyl-Inositol 3-Kinase (PI3K/Akt) signaling pathways, protecting tumor cells from apoptosis [4,44]. Microenvironmental acidity is a simple but highly efficient chemoresistance mechanism obtained by extracellular protonation and/or sequestration in acid vesicles. In fact, the extracellular pH in normal conditions is basic, while in malignant tumors it is acidic. Cancer cells have developed the ability to survive in an acid-hypoxic environment, which is not permissive for normal cells. Several studies have shown that this condition is exploited by cancer cells to compromise the absorption of weakly basic drugs and, consequently, their cytotoxic effect on tumors [41]. In addition, TME adipocytes can be reprogrammed by MM cells and transformed into different profiles with distinctive characteristics. It has been hypothesized that MM cells may modulate lncRNA enrichment in adipocyte exosomes and, in turn, adipocyte-derived exosomal lncRNAs inhibit chemotherapy-induced apoptosis in MM cells [39].

## 4. Tumor Microenvironment

Nowadays, cancer research is mainly focused on understanding the complex relationship between the tumor and its microenvironment; a greater understanding of these interactions, in fact, would lead to the development of new therapeutic strategies to overcome DR [11,16]. Tumors develop in complex and dynamic microenvironments that interact through the lymphatic and circulatory systems, and can influence their growth and spread [11,56]. The TME is defined by the set of molecules, cells, tissues, and structures that surround the tumor. The TME, therefore, consists of a diversified cellular and acellular environment in which cancer stem cells (CSC) can grow and develop, thanks to stromal and immune cells that are used by the tumor to create a self-sustaining surrounding environment [57]. Indeed, several studies have demonstrated that tumor growth and the process of metastasis are influenced by multiple bidirectional exchanges between cancer cells and their microenvironment [58]. In hostile conditions—such as hypoxia, lack of nutrients, acidic environment, and chemotherapy—cancer cells can, in fact, “recruit” non-malignant cells nearby, and use them to their advantage to create a “hospitable environment” promoting tumor growth and spreading [58,59,60]. As such, during tumor growth, the “seed” of the tumor develops together with the “soil”, the surrounding microenvironment; this hypothesis, which has been demonstrated by several studies, was formulated for the first time by Stephen Paget and is known as the “seed and soil theory”, describing for the first time the importance of the TME in the development of cancer [61,62]. The TME consists of a non-cellular component, the extracellular matrix (ECM) and a cellular component (tumor, stromal, immune, inflammatory, endothelial cells) that communicate through a dense network of soluble factors (SF) (cytokines, chemokines, growth factors, etc.) [63,64] or mediators promoting horizontal gene transfer (such as cell-free DNA, apoptotic bodies, circulating tumor cells, miRNA, and exosomes) [65,66,67]. Cells “recruited” by the tumor, using these mediators, promote tumor growth and spreading, even in hostile conditions through mechanisms such as neo-angiogenesis, ECM remodeling, cell migration, suppression of the immune system, and DR [56,68,69]. Tumor-associated stromal cells (TASCs) have different cellular origins and at least six different cell types have been identified: fibroblasts, adipocytes, pericytes, macrophages, mesenchymal stem cells (MSCs) of BM, and immune cells [65]. The progression of the tumor, within the TME, is facilitated by an evident cellular trans-differentiation: from stromal cell to stromal cell, but also from tumor cell to stromal cell [70,71]. These stromal cells have a key role in tumor growth, promote uncontrolled cell proliferation and metastasis, and can increase resistance to drug treatments [72,73,74]. Among the cells residing in the TME, cancer-associated endothelial cells (CAECs) are involved in the formation of new tumor blood vessels, thus promoting tumor growth by increasing the nutritional support associated with neo-angiogenesis [75]. Immune cells, such as tumor-associated macrophages (TAMs), can promote the escape of cancer cells to distant sites through the circulatory system and are involved in immune editing, in MDR mechanisms, and in the suppression of the antitumor immune response [75,76,77]. Tumor-associated fibroblasts (TAFs) and cancer-associated fibroblasts (CAFs) are the most abundant stromal cells within the TME [78], and are involved in many stages of tumor development: they are involved in ECM remodeling [79]; promote neo-angiogenesis through the secretion of SF, and the subsequent trans-differentiation of pericytes into endothelial cells and then into TASCs [80]; have a pro-inflammatory mode of action by stimulating the production of cytokines [81]; and are able to antagonize therapeutic treatments [82]. The continuous replication of cancer cells alters oxygen levels resulting in hypoxia that increases the release of extracellular vesicles (EVs), such as exosomes, by cancer cells [9,13,83]. Several studies have recently focused on the role these microvesicles in tumor–stroma interactions and their production by TAFs [56]. In response to hypoxia, cancer cells adapt to the hypoxic environment to survive, through a variety of cellular mechanisms analyzed below [52].

### 4.1. Tumor Microenvironment in Hematopoietic Tumors

In solid tumors, the TME is an appropriate and functional environment for the survival, growth, and propagation of CSCs through the intermediation of growth factors produced by inflammatory cells, immune tolerance, and the development of new blood vessels essential for the nutrition of cancer cells [84]. Blood cancers, on the other hand, are characterized by a specialized tissue microenvironment: the BMME or BM “niche” consisting of the BM, secondary lymphoid organs, hematopoietic stem cells, and accessory stromal and T cells that promote malignant growth and induce DR [85]. BMME consists of a cellular and a non-cellular compartment, the latter represented by ECM proteins and SF (cytokines, chemokines, and growth factors). The cellular compartment, on the other hand, consists of hematopoietic cells (T-cells, B-cells, natural killer cells (NK), and myeloid cells) and non-hematopoietic cells (osteoblasts, osteoclasts, fibroblasts, endothelial cells, pericytes, mesenchymal stromal cells) [44]. A better understanding of the BM niche remodeling in leukemia, lymphoma, and myeloma can lead not only to a better understanding of the pathogenesis of these cancers but would also allow for a faster and more accurate diagnosis and, therefore, more targeted treatments in order to avoid relapse. [86]

#### 4.1.1. Leukemic Bone Marrow Niche

Leukemia cells are able to “hijack” the BM niche and create, through “remodeling”, a microenvironment favorable to their own growth. In this way, malignant cells become leukemic stem cells (LSCs) that grow more favorably than existing hematopoietic cells by exploiting the same molecular mechanisms of hematopoietic stem cells (HSCs) [86,87]. By hijacking the BM niche, malignant cells can ensure disease progression by suppressing the normal hematopoiesis process, evading tumor immunity, and promoting neo-angiogenesis [88]. In addition to the hematopoietic niche, leukemia cells interact with non-hematopoietic components—such as mesenchymal and vascular endothelial cells, osteoblasts, adipose cells—as well as the immune system, and the sympathetic nervous system [86]. Specifically, LSCs maintain and enhance their functionality by inhibiting macrophages [89], reducing the number of osteoblasts [90], interfering with the normal functions of the sympathetic nervous system [91], and by using the fatty acids of the adipocytes to generate useful energy for their proliferation [92]. Several studies have shown that exosomes also play a key role in the development of hematological malignancies. These vesicles are able to act as mediators of communication between malignant cells and the surrounding microenvironment [93].

#### 4.1.2. Lymphomagenesis and Tumor Microenvironment

Several studies have now amply demonstrated that the onset and progression of lymphoma requires reciprocal and dynamic crosstalk between the cancer cells and the surrounding microenvironment [94]. Lymphomagenesis, therefore, is not an autonomous process, but is closely associated with the presence of a tumor “niche” that has a crucial impact on the development and progression of lymphoma. The TME of B-cell lymphomas includes BM and lymphoid organs (lymph nodes and spleen) and is significantly different from the tumor niche of solid tumors, but also from other hematological tumors [95].

The lymphoma niche consists of a non-cellular component (extracellular matrix, soluble factors, cytokines, and chemokines) and a cellular component consisting primarily of a variable number of immune cells (T- and B-cells, dendritic cells, antigen-presenting cells, and macrophages), mesenchymal stem cells, and stromal cells (fibroblastic reticular cells and mesenchymal stromal cells) [96]. MSCs play a critical role in shaping lymphoma TME; they regulate tumor engraftment, neo-angiogenesis, and are often involved in DR mechanisms [97,98]. Macrophages that are present in the lymphoma niche are named lymphoma-associated macrophages (LAM) and, by contributing significantly to tumor growth, have a fundamental importance in the pathogenesis and prognosis of lymphoma [95]. Numerous intratumoral T-cells are also present in the TME of the lymphoma, representing a very heterogeneous group of immune cells composed of various subpopulations: regulatory T cells (Treg), helper T cells (Th), CD8 + T cells, and follicular helper T cells (TFH) [99]. Although the specific role of these T cells in tumorigenesis is very complex and not yet entirely clear, several studies have shown that they not only induce tumor progression through the release of cytokines and/or direct contact with them, but they also play a key role in immunosuppression, and activation of the antitumor immune response—thereby promoting an immuno-proprietary microenvironment [57,100].

#### 4.1.3. The Tumor Microenvironment in Multiple Myeloma

The BMME also plays a key role in the pathogenesis of multiple myeloma (MM). The transformation of normal plasma cells (PCs) into monoclonal malignant PCs (MM cells) is associated, in fact, not only with oncogenic events and molecular changes (genomic/chromosomal alterations), but also with the dynamic interaction between MM cells and the surrounding microenvironment [101]. The consequence of these interactions, between malignant cells and microenvironmental cells, is the formation of specialized niches or “MM niches” that provide a permissive environment for survival and growth of cancer cells [102]. In these niches, MM cells proliferate in an environment protected from apoptotic stimuli and, by acquiring the DR phenotype, become resistant to therapeutic treatments [103]. The osteoblast niche, localized in the endosteum, is involved in hematopoiesis and in the regulation of the quiescence and self-renewal processes of HSCs. MM cells are able to hinder the formation and differentiation of osteoblasts by binding and degrading osteoprotegerin (OPG). The subsequent activation of osteoclasts (which are no longer inhibited by OPG) will result in bone loss and osteolytic lesions [104,105]. The vascular niche is, instead, involved in the process of neo-angiogenesis; MM cells induce pro-gene-angiogenic genes and promote, hereby, the vascularization of the TME and the growth of endothelial cells by increasing the secretion of growth factor, leading to the development and progression of MM. In fact, in the body fluids of patients suffering from this cancer form, higher concentrations of exosomes were found, when compared to healthy subjects, suggesting an involvement of these vesicles in MM pathogenesis and their possible use as biomarkers for early diagnosis [106].

## 5. Hypoxia and Tumor Microenvironment

**Figure 1:**
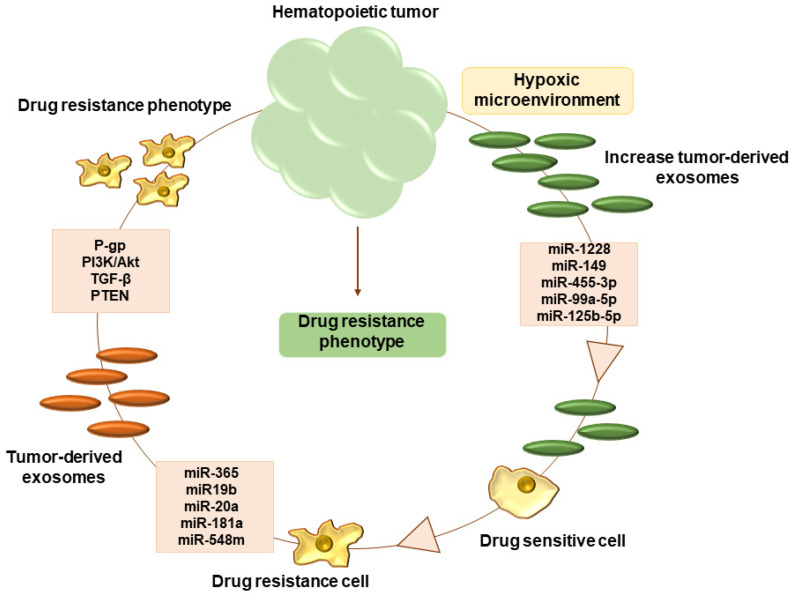
Hypoxic microenvironment of hematopoietic tumor, increase exosome release. Exosome content with different miRNA involved in acquisition of drug resistance phenotype through different pathway.

Hypoxia is a common phenomenon observed in many malignant tumors and is involved in DR. [107]. Cancer cells encounter, in the microenvironment, low oxygen levels due to insufficient oxygen flow and physiological abnormalities in tumor vessels, resulting in normoxic, hypoxic, and even necrotic regions [108]. In most solid tumors, the average oxygen concentration is close to 10 mmHg, while in other tissues it reaches 40 and 60 mmHg [109]. In fact, rapid tumor proliferation leads to a drop in normal oxygen levels from 2–9% to hypoxic levels below 2% [110]. Hypoxia is an often-transient phenomenon, which occurs in microscopic sites within the TME; it represents a condition of oxygen deficiency and is a common feature of many solid tumors related to tumor development, resistance to therapy, and mortality [111]. In response to hypoxia, in order to survive, cancer cells adapt to this environment through a variety of cellular mechanisms that induce changes in gene and protein expression, with important physiological repercussions. In the hypoxic region, the adaptations of the tumor cells are different: in the hypoxic environment, the activation of the HIF-1α factor increases the activity of Twist and Snail, two transcription factors that reduce the expression of E-cadherins, and that promote the epithelium–mesenchyme transition (EMT). The EMT is a mechanism involved in the processes of resistance to chemotherapy [112]. Hypoxia is also associated with a reduction in extracellular pH in TME which induces DR by inhibiting the humoral and cellular immune response, and by up-regulating the transporter protein P-gp. Another adaptation is the Warburg effect, enhanced by the expression of Pyruvate Kinase M2 (PKM2), and is able to induce resistance to cisplatin in CAFs following their reprogramming [55]. It has been widely demonstrated that tumor-associated cells secrete more exosomes than normal cells [113]; a number twice as large as for the amount contained in healthy human blood has been estimated [114,115]. All these results indicate that the effects caused by hypoxia on tumors can be observed also in an increasing number of cancer cell exosomes, which can carry signals to recipient cells (ex. protein, miRNA, enzymes, lncRNA, transcriptional factor etc.) [113]. The increase in exosome number could explain the compensation, both in the demand of tumor cells (nutrient, vessel, replication) and the tumor microenvironmental modification (hypoxia), thanks to the different compositions of the exosomes, in particular miRNA. However, the detailed mechanism by which hypoxia increases exosome release from cancer cells is still not well understood. Therefore, it could be conceivable that more exosomes are needed to meet cell communication needs in cancer, due to the complicated hypoxic environment that develops in tumors. Recent studies report that hypoxia stimulates the increase in exosome levels, thus facilitating the intercellular communication of the tumor at a distance [107]. In addition, exosomes also play a fundamental role in TME: they are involved in the inflammatory process, neo-angiogenesis, and metastasis [116]. In Figure 1 we summarize the mechanism of DR acquisition in a hematological tumor.

## 6. Exosome in Tumor Microenvironment

Several studies identified exosomes as important regulators of DR mechanisms in tumor treatment [117]. To further demonstrate the relationship between exosomes and miRNA, we discuss the exosome biogenesis and their composition, specifically miRNA involved in the acquisition of the drug resistance phenotype. The term exosome was first described in lattices during erythrocyte maturation by Rose M. Johnstone et al. in the 1980s [118] and over the years it has often occupied a marginal niche, due to the supposed role of exosomes as cellular “trash cans” [119]. They are membrane-coated vesicles, which are a subset of intracellular vesicles (IVs) with a diameter of 40–100 nm, secreted by many cell types [120] into the ECM via multivesicular bodies (MVBs), including cancer cells. They contain proteins, mRNAs, miRNAs, transcription factors, lipids, and other biologically active constituents and exert their role in intercellular communication by mediating the exchange of these molecules between cells, thus modifying the bio-properties of the receiving cells. Therefore, given the scientific evidence of the role and importance of exosomes, they have emerged as essential actors in extracellular communication [121,122,123]. Exosomes are of particular interest in biological research because their unique biogenesis involves distinct intracellular regulatory processes that give them specific loads and, therefore, different biological functions [115,124]. The transfer of these specific loads to recipient cells induces several phenotypic changes [125]. Exosome-mediated cellular communication has recently been identified as a mechanism involved in DR [126,127] through directly exporting drugs, inducing inactivation, and supplying non-coding proteins and RNAs, significantly to the resistance against the latter [128,129]. Thanks to their intrinsic capacity to carry proteins, lipids, and nucleic acids, they are also clinically significant diagnostic biomarkers and therapeutic targets and represent a vehicle for the administration of anticancer drugs [16].

### 6.1. Exosome Biogenesis

The biogenesis of the exosome comprises three different stages, including: (i) formation of endocytic vesicles through plasma membrane invagination (early endosome); (ii) formation of MVBs containing intraluminal vesicles (ILV) generated by the budding towards the inside of the endosomal membrane with the cytoplasmic constituents; (iii) fusion of MVBs with the plasma membrane, and the extracellular release of ILV as exosomes [130,131,132]. Furthermore, MVBs can be degraded through fusion with lysosomes or autophagosomes [133]. A multitude of proteins are involved in the maturation of MVBs and ILV, including the endosomal sorting complexes required for transport (ESCRT) proteins, which consist of four different protein complexes, ESCRT-0, -I, -II, and -III [130,134,135]. In addition to ESCRT proteins, the X protein interacting with the apoptosis linked gene 2 (ALIX) [136], vesicle trafficking 1 (VTA1) protein [137], the soluble *N*-ethylmaleimide-sensitive factor attachment protein receptor (SNARE) proteins [138], and guanosine triphosphatase (GTPases) are also considered important actors in the biogenesis and secretion of exosomes [139]. There are several ways in which exosome-induced DR can be mediated [8], such as through direct drug export, drug efflux pump transport, and miRNA signaling [135,140]. Once released by cancer cells, exosomes can directly induce DR in the tumor- surrounding environment where sensitive cells become resistant, or they can interact and deliver miRNA to cells of the TME hereby modulating the DR response [141]. In the TME, tumor-derived exosomes mediate the communication between tumor and stromal cells, thus contributing to therapy evasion and tumor growth [126]. Recently, tumor-derived exosomes have been reported to play a substantial role in the differentiation of TME fibroblasts into CAFs, promoting tumor growth, pro-angiogenic, invasive, and drug-resistant phenotypes [142,143,144]. CAFs are the most abundant type of stromal cell in the TME and exhibit the same characteristics as myofibroblasts found during the wound healing process [145]. According to Webber et al., transforming growth factor beta-1 (TGFβ1), transported by cancer exosomes, is required to activate the tumor-promoting stroma [146]. In fact, recent studies revealed a relationship between exosomal miRNAs derived from cancer cells and CAFs differentiation in many types of cancer [147]. Recent findings highlight the role of miRNAs released by tumor-associated exosomes that act as messengers in the TME, capable of inducing the differentiation of fibroblasts into CAFs [55]. Thus, released miRNAs directly act on the induction pathways of DR.

### 6.2. Exosomes in Leukemia

Recent studies revealed that exosomes participate in the development of leukemia, and play important roles in its diagnosis and treatment by influencing cell proliferation and apoptosis, regulating the BM microenvironment, promoting angiogenesis, inhibiting hematopoiesis, and also influencing DR. Leukemia-derived exosomes present leukemia-related antigens to target cells, promote the proliferation of leukemic cells, help these cells escape immunity, and protect them from the cytotoxic effects of chemotherapeutics related to metastasis [148]. Leukemic cells have a mechanism of exosome-mediated angiogenesis that allows them to proliferate more actively, and to lay the groundwork for spreading to distant organs.

Recently, the evasion of immune responses by tumors has been attracting significant attention by the development of new therapies, such as immunotherapy. As a result, the mechanism underlying immune response evasion in leukemia is becoming clearer, with exosomes having an important role in it [149,150]. Hong et al. reported that the serum of patients with leukemia contained more exosomes compared to healthy subjects, and it was particularly rich in TGF-β1, which plays an important role in the immune evasion of tumors [151]. TGF-β1 in leukemic cell-derived exosomes suppresses the tumor-recognition function of NKG2D expressed on NK cells and cytotoxic T cells [152].

#### 6.2.1. Exosomes in Chronic Myeloid Leukemia (CML)

The BM of CML patients is known to be hypoxic, although angiogenesis is marked [153,154]. It has been reported that the release of exosomes by chronic myeloid leukemic cells is associated with a mechanism that promotes angiogenesis in human umbilical vein endothelial cells (HUVECs) [155]. IL-8, which, like exosomes, is secreted by CML cells, stimulates HUVECs in order to increase the expression of intercellular adhesion molecule-1 (ICAM-1) and vascular cell adhesion molecule-1 (VCAM-1). This is one example of how CML cells can stimulate their growth by changing their preferred BMME through exosome-mediated angiogenesis.

In addition, exosomes secreted by CML cells can be taken up by endothelial cells and can promote endothelial cell tube formation. Exosomes from K562 cells induced dasatinib-sensitive Src phosphorylation, and activation of the downstream Src pathway proteins in endothelial cells. Dasatinib activity may be greater than imatinib (IM) activity due to the involvement of Src in both leukemia cells and the angiogenic microenvironment [154]. Furthermore, a study from Tokyo Medical University found that pre-miR-92a derived from K562 exosomes could reduce the expression of the target gene integrin a5, thereby enhancing endothelial cell migration and tube formation. miR-210 from CML exosomes has been shown to interplay with the target gene Ephrin-A3 and play an important role in the regulation of angiogenesis and vascular endothelial growth factor (VEGF) signaling. These findings demonstrate how leukemic cells may convey signals to the microenvironment and highlight the therapeutic potential of using exosome-derived miRNAs in combination with currently available VEGF inhibitors [156].

#### 6.2.2. Exosomes in Acute Lymphoblastic Leukemia (ALL)

In the treatment of ALL, the use of central nervous system (CNS)-transferring chemotherapy as prophylaxis has been incorporated into treatment regimens. The CNS remains a critical site of invasion and recurrence that determines the prognosis in ALL. Ichiko et al. reported that in B-cell ALL, leukemic cell-derived exosomes containing IL-15 are taken up by astrocytes and brain vascular endothelial cells, leading to the production of VEGF-AA by astrocytes and to the disruption of the blood–brain barrier, resulting in central nervous system infiltration [157].

#### 6.2.3. Exosomes in Acute Myeloid Leukemia (AML)

Even in the case of AML, exosomes support their growth by acting as messengers between tumor cells and the microenvironment, and by inducing oncogenic factors such as c-Myc [25]. Thanks to the studies by Wang et al. in 2019, regarding HUVECs, it has been demonstrated that exosomes containing VEGF/VEGFR secreted by AML can induce glycolysis, leading to vascular remodeling and the acquisition of chemoresistance [158]. Furthermore, serum of AML patients is known to be rich in exosomes, which can suppress the immune functions of NK cells by reducing their cytotoxicity. These exosomes contain elevated levels of TGFβ1, a potent immunosuppressant for NK that induce down-regulation of NKG2D, a transmembrane protein/lectin-like type c receptor expression [159]. Based on previous studies, NK cell activities such as cytokine production and intracellular signaling are reduced in AML. NK cell dysfunction could be a causative factor for AML initiation, development, progression, or relapse [160].

### 6.3. Exosomes in Lymphomas

The malignant progression of lymphoma is complicated, and initially most studies that aimed to elucidate the mechanism of B-cell lymphomagenesis were focused on the aberrations in primary tumor cells. Nowadays, instead, there is a growing interest in the role of the TME, and the success of immunotherapies against malignant lymphoma confirms its importance [161]. Investigations on the TME have revealed that it contributes not only to tumorigenesis, but also to the maintenance of cancer stemness and resistance to treatment [162]. In particular, the extent of infiltration of microenvironmental cells, including lymphocytes and macrophages, is associated with clinical outcomes [35,161].

Regarding the development of a putative therapy based on B-cell lymphoma exosomes, Aung et al. [163] demonstrated that these exosomes, carrying CD20 and lysosome-related organelle-associated ATP Binding Cassette Subfamily A Member 3 (ABCA3), bound to the therapeutic anti-CD20 antibody and the consumed complement, hereby impaired antibody-dependent cell-mediated cytotoxicity (ADCC) and protected cancer cells from antibody attack. In addition, they also reported that, in patients treated with the therapeutic antibody for B-cell lymphoma, more than one-third of plasmatic rituximab was bound to exosomes. In addition, they observed a significant improvement in the effect of rituximab against in vitro lymphoma cell lines and autologous cancer cells due to the removal of exosomes from plasma samples [95].

### 6.4. Exosomes in Multiple Myeloma (MM)

Even in the case of MM, exosomes play a primary role and participate in the cross-talk between myeloma cells and nonmalignant components of the in vivo environment [43]. The interplay between bone marrow stromal cells (BMSCs) and MM cells results in the secretion of growth factors, cytokines, and extracellular vesicles, thus playing a crucial role in MM pathogenesis.

Although BMSC-induced MM growth and survival promotion has been investigated, the role of BMSC-derived exosomes in these actions remains unclear. Wang et al., 2014 investigated, in the 5T33MM mouse model and human samples, the effect of BMSC-derived exosomes on the viability, proliferation, survival, migration, and DR of MM cells [164].

Btz, lenalidomide, and thalidomide are known as clinically relevant drugs and are widely used in the treatment of patients with MM; many patients, however, continue to relapse and become resistant to these drugs. The BMME and the interaction between BMSC and MM cells are known to contribute to DR of these cells through cell-to-cell adhesion and secretion of soluble factors by BMSCs. As a result, several DR and cell survival related pathways, such as Notch1, signal transducer and transcription activator, Akt [102] and NF-κB, are activated to protect MM cells from death. Wang et Al, 2014 [164], examined the effect of exosomes on Btz-induced cell death. They demonstrated that Btz promotes MM cell death through induction of cleavage of caspase-9, caspase-3, and PARP (ADP-ribose polymerase). Moreover, both murine and human exosomes were found to inhibit these effects to protect MM cells from apoptosis. These results reveal, for the first time, a novel mechanism in the BMSC-induced DR of MM cells to Btz via exosome secretion [102,164].

## 7. Exosome and miRNA Regulation in DR

As we described previously, hypoxia is a common phenomenon observed in many malignant tumors and is involved in different adaptative mechanisms of tumor cells, exosome biogenesis, and miRNA regulation. Several studies have also shown that concentrations of circulating exosomes and exosomal miRNAs are elevated in patients with lung cancer, as compared to healthy controls [165]. Although the mechanism is currently unknown, miRNAs play a fundamental role, as they can activate changes in gene expression [166]. Additionally, a growing number of studies have associated microRNAs to DR, demonstrating their regulatory role even in the therapy response of hematological tumors [4].

### 7.1. Exosomal miRNA: Biogenesis

miRNAs are a class of small non-coding RNAs, typically 19–25 nucleotides long, which mediate post-transcriptional gene silencing by binding to the 3′-UTR region or to the Open Reading Frame of the target mRNA [8]. Therefore, miRNAs have not only the remarkable ability to regulate different biological processes—such as proliferation, angiogenesis, and metastasis—by altering gene expression, but also to remodel the TME in order to signal to the immune system [167]. It is also known that changes in the expression of miRNAs, during chemotherapy treatments, have been proposed as a tool to monitor the efficacy of the therapy and the risk of relapse [168]. At the same time, the TME modulates the expression of miRNAs by releasing SF (cytokines and growth factors) by both the tumor and non-tumor cells, or by the presence of mechanisms such as hypoxia, starvation, and pH alterations, as described above. A sequence of two 4-nucleotide sequences, recognized by the RNA binding protein Heterogeneous Nuclear Ribonucleoproteins A2/B1 (hnRNPA2B1), is used for the sorting of miRNAs in exosomes, as indicated by data in the literature. A study by Wang et al. has shown that, in hypoxic conditions of the TME, exosomes relate to transformation of the tumor phenotype by the transfer of miR-193-3p, miR-210-3p, and miR-510-5p [19] and are capable of activating the transition between epithelium and mesenchyme—one of the mechanisms used for DR [125]. In Table 1 we summarize miRNAs and the pathway that will be discussed in the following paragraph.

### 7.2. Exosomal miRNA: Mechanism and Action in Solid Tumor

Exosomes released by drug resistant cancer cells typically have a different cargo than exosomes released by sensitive cancer cells, hereby mediating the horizontal transfer of DR traits from drug-resistant cancer cells to drug-sensitive ones. The different exosomal miRNAs exert their role in DR through the following mechanisms: I) by alteration of the drug efflux pump, II) increased resistance to apoptotic stimuli, and III) modulation of the tumor microenvironment [5,50].

#### 7.2.1. Alteration of Drug Efflux Pump

Many studies suggested that exosome secretion acts as a signal to change the sensitivity of tumors to chemotherapy drugs and tumor progression. Meanwhile, exosomes can expel a variety of compound drugs out of tumor cells, reduce tumor cells’ DR, and thus lead to the failure of chemotherapy [178]. Levchenko and colleagues demonstrated that the expression of exosomal protein P-gp is regulated by miR-451 and miR-27a. They can maintain a prolonged expression of the P-gp proteins, with consequent upregulation, which contributes to the activation of the nuclear factor kappa-light-chain-enhancer of activated B cells (NF-kB). These cells are known to be involved in DR mechanisms [19], through the alteration of drug efflux pumps [179]. In fact, P-gp, the most important drug transporter, can induce an acquired resistance phenotype associated with miRNAs, through blocking apoptosis by increasing the expression of the MDR1 gene, which encodes P-gp [180].

#### 7.2.2. Increased Resistance to Apoptotic Stimuli

Overall, miR-21 is a key regulator of the oncogenic processes that promote cell survival, and the formation and activation of CAFs [8] by regulating TGFβ1 signaling [181]. Isolated exosomes derived from hepatocellular carcinoma (HCC) patients can convert normal hepatic stellate cells to CAFs via miR-21, which downregulates the phosphatase and tensin homolog (PTEN) tumor suppressor gene, and consequently upregulates the PI3K/Akt signaling pathway [182]. The Yan-Ran group has instead shown that miR-21, released from gastric tumor cells, promotes resistance to apoptosis through the PTEN/PI3K/Akt pathway; it is known for its role in the regulation of various biological processes such as transcription, protein synthesis, metabolism, autophagy, proliferation, apoptosis, angiogenesis, and migration [183]. The regulation of miRNAs involves a loss of function of PTEN, which acts as a gatekeeper of the pathway, with a consequent increase in the invasion capacity and aggressiveness of the tumor. On the other hand, a gain of function of PI3K and Akt promotes survival and anti-apoptotic mechanisms [184]. miR-21, released from exosomes derived from cells resistant to cisplatin, induces resistance to sensitive cells by acting on the pathway of phosphatases and the homolog of tensin (PTEN) to regulate the mechanism of programmed cell death [117]. Fu et al. demonstrated that MDR liver cells transfer miR-32-5p to sensitive cells by exosomes, which in turn activates the PI3K pathway through the Akt pathway, capable of inducing resistance [185].

#### 7.2.3. Modulation of the Tumor Microenvironment

As we have described, this bidirectional communication between the tumor cells and the fibroblasts reprogrammed in CAFs, through the exosomes, allows for the creation of an optimal microenvironment for tumor growth, giving a more aggressive phenotype to tumor cells; thus, allowing them to adapt to the “new” microenvironment [186]. A further study showed that miR-222, released from exosomes in lung cancer cells, is correlated with a high tumor aggressiveness, as these miRNAs act on the downregulation of the target gene PDLIM2, which in turn activates the nuclear factor NF-kB [187]. Further studies have been carried out on miR-1246, which is highly present in solid tumors, and is found in the microenvironment and released by exosomes. The overexpression of Caveolin 1 (Cav1), the direct target of miR-1246, sensitizes cells to paclitaxel, by reducing the levels of the p-gp protein (MDR1) [188]. Furthermore, a study using exosomal human melanoma miRNAs showed that miR-155 and miR-210 induce metabolic changes in adult human fibroblasts to increase aerobic glycolysis, promoting a pre-metastatic microenvironment [189]. In particular, miR-155 and miR-210 are referred to as oncogenic miRNAs that drive resistance to therapy in different types of cancer [190,191,192]. While cancer-derived exosomal miRNAs can promote CAFs differentiation, CAFs-released exosomal miRNAs in the TME play an important role in therapy resistance. Next-generation sequencing technology demonstrated that exosomal transfer of miR-21, from CAFs to ovarian cancer cells, inhibited apoptosis and promoted resistance to treatment with different types of drugs. [8]

### 7.3. Exosomal miRNA in Hematological Tumor

miRNAs can be transferred from tumor cells to adjacent cells in their cell-free circulating exosome [193]. miRNA expression has been widely associated with different diseases, including several cancers such as hematological malignancies, through the targeting of mRNAs coding for proteins influencing drug-resistance (such as drug efflux pumps, increased resistance to apoptotic stimuli, and modulation of the tumor microenvironment) [194]. Table 2 summarizes the principal action type of exosomes and related miRNA, with their specific mechanisms.

#### 7.3.1. miRNA and Leukemia: Chronic Myeloid Leukemia (CML)

In CML, the majority of patients can be treated with TKIs, such as IM, but some patients will develop DR. Studies have identified exosomes as promoters of DR in cancer [195]. Jaiswal et al. demonstrated that several miRNAs (miR-1228*, miR-1246, miR-1308, miR-149*, miR-455-3p) were more abundant in the exosomes of the ALL cell line CCRF-CEM (designated CEM for simplicity), compared to healthy donor cells. The same miRNAs were found to be significantly increased in drug-sensitive cells upon co-culturing with drug-resistant exosomes [169], confirming the role of exosomes in tumor progression. Min et al. demonstrated that IM-sensitive CML cells exhibited DR after having been incubated with exosomes derived from IM-resistant CML cells. They identified a differential miRNA expression profile in exosomes, deriving from either IM-resistant or IM-sensitive CML cells. Moreover, they observed the highest miR-365 expression level in exosomes of IM-sensitive CML cells, and identified that exosomes mediated the transfer of miR-365 between IM resistant and sensitive CML cells, and that miR-365 induced DR by inhibiting expression of pro-apoptosis proteins BAX and Cleaved Caspase-3 in IM-sensitive CML cells [170]. Additionally, another study confirmed that the transfer between resistant and sensitive CML cells of the drug resistant phenotype is mediated through exosomes [171]. Exosomes deriving from resistant cells, containing miR-27a, miR-451, and miR-21, have been shown to be related to P-gp expression and can also contribute to chemotherapy resistance in tumor cells. Another study suggests that exosomes released by IM-resistant CML cells (K562/G01) were internalized by sensitive CML cells (K562), thus horizontally transferring miR-365 that confers DR to the recipient cells [170].

##### Acute Myeloid Leukemia (AML)

The upregulation of miR-7977 in AML exosomes was found to have a role in the disruption of normal hematopoiesis, and in the aberrant production of hematopoietic growth factors [172]. P-gp can be expressed on cancer cells of AML patients and contributes to resistance to anthracyclines in vitro. The study of Bouvy demonstrated that exosomes from the drug-resistant HL-60 cell line (HL-60/AR) can interact with their sensitive (HL-60) counterpart cells and enable resistant cells to transfer their drug-resistance phenotype to sensitive cells [173]. In particular, two miRNAs, miR-19b and miR-20a, were found to be more expressed (> 4-fold change) in exosomes of sensitive cells as compared to resistant cells. These increased P-gp expression, targeted PTEN, and decreased the activation of the PI3K/Akt signaling pathway; this signaling pathway is often constitutively activated in AML [173].

##### Chronic Lymphocytic Leukemia (CLL)

CLL-derived exosomes have been demonstrated to deliver miRNAs to stromal cells that acquired features of cancer-associated fibroblasts, promoting CLL progression. In particular, miR-146a was specifically enriched in CLL exosomes as compared to a healthy subject’s cells (ratio 15:1) and was able to induce activation of the Akt pathway and NF-kB [173].

#### 7.3.2. Exosomal miRNA and Lymphoma

Follicular dendritic cells (FDCs) are stromal cells that support lymphoma progression, and their adhesion to NHL cells is associated with DR [174]. Lwin et al. showed that NHL cell adhesion to FDC induced the upregulation of miR-181a, through B-cell lymphoma-2 (BCL-2)–interacting mediator of cell death (BIM) downregulation and, consequently, protected lymphoma cells from drug-induced apoptosis [174]. The same group discovered that miR-548m downregulation, and subsequent histone deacetylase 6 (HDAC6) overexpression, enhances the adhesion of NHL to lymphoma stroma cells, promoting sustained c-Myc activation. Interestingly, c-Myc is a target of miR-548m, forming an amplification loop in these cancers. Both mechanisms, regulated by the tumor microenvironment, suggest that alterations in specific miRNA levels lead to DR and lymphoma survival [175]. Additionally, in NHL and diffuse large B-cell lymphoma (DLBCL), miR-99a-5p and miR-125b-5p were found significantly upregulated in exosomes from resistant cells, when compared to sensitive parental cells’ exosomes [176]. The expression levels of miR-99a-5p and miR-125b-5p were higher in exosomes from the serum of chemo-resistant DLBCL patients, as compared to the sensitive ones, thus being associated with a worse prognosis [176]. Additionally, it has been suggested that exosomal miRNAs could be used as predictive biomarkers of chemotherapeutic efficacy in DLBCL patients [176].

#### 7.3.3. Exosomal miRNA and Multiple Myeloma (MM)

Thanks to the novel therapy for MM patients using Btz, panobinostat, melphalan, and lenalidomide, the survival rate of these patients has significantly improved in the past years. The interaction between myeloma cells and the BMME plays a pivotal role in MM initiation, progression, and DR. BMME-derived exosomes antagonize Btz-induced apoptosis and decrease the viability of MM cells [40]. Tang and co. employed KEGG analysis on Btz-resistant MM cells and found four enriched terms associated with DR, including the mechanistic target of rapamycin (mTOR), and the cAMP and PI3K–Akt signaling pathways. Moreover, bone marrow stromal cell derived exosomes (BMSC) influence multiple signal transduction pathways, such as JNK and p38 MAP kinases (MAPK), p53, and Akt, that affect the survival of MM cells, thus stimulating MM-cell growth and inducing Btz resistance [40]. Their results also indicate that miR-627-3p and miR-642a-5p target the transcriptional factor SP9, and that these miRNAs may bind differentially expressed exosomal lncRNA involved in PI3K-Akt signaling pathways [40]. Zhang et al. analyzed the cargo of exosomes from the plasma of MM patients treated with Btz and identified several miRNAs [43]. Specifically, they found exosomes with significantly increased internal RNAs and a downregulation of miR16-5p, miR-15a-5p, miR-20a-5p, and miR-17-5 in exosomes from patients resistant to Btz, as compared to patients sensitive to Btz. This suggests the use of exosomal miRNAs as DR biomarkers for MM [43]. Moreover, Zhao et al., knowing of the correlation between miR-30 and B-cell lymphoma 9 (BCL9), observed another correlation between MM cell lines and cells from MM patients. In fact, H929 cells (MM cell lines) co-cultured with BMSCs showed miR-30 downregulation, associated with an enhanced expression of BCL9, which is a transcriptional coactivator of the Wnt signaling pathway, known to promote multiple myeloma cell proliferation and DR [177].

## 8. Conclusion and Perspective

DR is the biggest challenge in the fight against cancer. The study of the microenvironment, of all its components, and of the integration of the pathways, is of fundamental importance. As we have discussed here, tumor cells can adapt to new environments, becoming more aggressive, and evading control mechanisms, in a context that is already finely regulated with the aim of guaranteeing their growth and survival. Indeed, one could think of counteracting the pro-tumorigenic signals that contribute to DR, by engineering the miRNAs and the contents of the exosomes in order to interfere with their traffic [196]. Through transporting the exosomal cargo, such as miRNA, drug sensitivity can be modulated via multiple mechanisms [18]. In fact, recent studies have also analyzed the other side of exosomes, namely their ability to transfer and communicate within the microenvironment, which could also prove to be a strategy for administering drugs. At present, from a pharmacological point of view, the biggest obstacle is to find a safe and effective RNA delivery system in the cytoplasm. Lipids are the vehicles currently used for the transport of RNAs [18]. For this purpose, there are many ongoing studies on the analysis of the content of exosomes [197]; Sphingosine-1-phosphate and sphingosine kinase 2 (Sphk2) may play a role in regulating the cargo loading into the exosomes [18]. Additionally, engineered exosomes called Synthetic Multivalent Antibodies ReTargeted Exosomes (SMART-Exos) can express monoclonal antibodies that target T cells and tumor cells, respectively [18]. Therefore, engineered exosomes and defined cargo may overcome the DR, and once discovered, pro-tumor signals can be blocked, and exosomes can also prove to be supportive, both in pharmacological treatment and in use as biomarkers for the progression of some tumors.

## Figures and Tables

**Table 1:** Principal miRNAs involved in drug resistance (DR) of hematological tumor.

Hematological Tumor	miRNA	Drug Resistance	Cell Type	Pathway	Ref.
**Chronic myeloid leukemia** **(CML)**	miR-1228 miR-1246 miR-1308 miR-149 miR-455-3p miR-365 miR-27a miR-451 miR-21	Imatinib Imatinib Imatinib Imatinib Imatinib Imatinib Resistance phenotype Resistance phenotype Resistance phenotype	CEM CEM CEM CEM CEM CML CML CML CML	Exosome increaseExosome increase Exosome increase Exosome increase Exosome increase Bax-Caspase3 P-gp P-gp P-gp	[169] [169] [169] [169] [169] [170] [171] [171] [171]
**Acute myeloid leukemia** **(AML)**	miR-7977 miR-19b miR-20a	Anthracyclines Anthracyclines Anthracyclines	HL-60/AR HL-60/AR HL-60/AR	P-gp PTEN and P-gp PTEN and P-gp	[172] [173] [173]
**Chronic lymphocytic leukemia (CLL)**	miR-146a	Resistance phenotype	CCL	Akt-NF-kB	[173]
**Lymphoma**	miR-181a miR-548m miR-99a-5p miR-125b-5p	Resistance phenotype Resistance phenotype Resistance phenotype Resistance phenotype	NHL NHL DLBCL DLBCL	Bim c-Myc-HDAC6 Exosome increase Exosome increase	[174] [175] [176] [176]
**Multiple myeloma**	miR-627-3p miR-642a-5p miR-16-5p miR-15a-5p miR-20a-5p miR17-5 miR-30	Bortezomib Bortezomib Bortezomib Bortezomib Bortezomib Bortezomib Resistance phenotype	LP-1 myeloma cells LP-1 myeloma cells U-226 myeloma cell U-226 myeloma cell U-226 myeloma cell U-226 myeloma cell H929 myeloma cell	SP9-PI3K/Akt SP9-PI3K/Akt Exosome increase Exosome increase Exosome increase Exosome increase BCL9-Wnt	[40] [40] [43] [43] [43] [43] [177]

**Table 2:** Principal type of action of exosomes and related miRNAs with their specific mechanism.

Type of Action	Name	Mechanism	Ref.
*Exosome increase*	miR-1228, miR-1246, miR-1308, miR-149, miR-455-3p	More abundant in the exosomes of ALL cell line	[169]
	miR-99a-5p, miR-125b-5p	Higher in exosomes from serum of chemo-resistant patients	[176]
*Drug resistance*	miR-365	Inhibiting expression of pro-apoptosis proteins BAX and Cleaved Caspase-3 in CML cells	[170]
	miR-27a, miR-451, miR-21	Increasing expression of P-gp	[171]
	miR-7977	Increasing expression of P-gp	[172]
	miR-19b, miR-20a	Targeting PTEN and decreasing activation of PI3K/Akt signaling pathway	[173]
	miR-146a	Activating Akt pathway and NF-kB in CLL	[173]
	miR-181a	Protecting lymphoma cells from drug-induced apoptosis through BIM	[174]
	miR-548m	Promoting c-Myc activation in lymphoma cells	[175]
	miR-627-3p, miR-642a-5p	Targeting SP9 and activation of PI3K-Akt signaling pathway	[40]
	miR-30	Enhancing expression of BCL9, a transcriptional coactivator of the Wnt signaling pathway	[177]
*Exosomal biomarker*	miR-15a-5p, miR-20a-5p, miR-17-5, miR-16-5p	Downregulation in exosomes from patients resistant to Btz compared to patients sensitive to Btz	[43]

## Data Availability

Not applicable.

## Abbreviations

ABCA3	ATP Binding Cassette Subfamily A Member 3
ABC	ATP-binding cassette
ALL	Acute lymphoblastic leukemia
AML	Acute myeloid leukemia
ADCC	antibody-dependent cell-mediated cytotoxicity
ADME	absorption, distribution, metabolism and elimination
ALIX	apoptosis linked gene 2
BCL9	B-cell lymphoma 9
BIM	B-cell lymphoma-2 [BCL-2]–interacting mediator of cell death
BM	bone marrow
BMME	bone marrow microenvironment
BMSCs	bone marrow stromal cells
Btz	bortezomib
CAECs	cancer-associated endothelial cells
CAFs	cancer-associated fibroblasts
CAV1	Caveolin 1
CFU	colony forming unit
CLL	Chronic lymphocytic leukemia
CML	Chronic myeloid leukemia
CNS	central nervous system
CSC	cancer stem cells
DLBCL	diffuse large B-cell lymphoma
DR	drug resistance
ECM	extracellular matrix
EMT	epithelium-mesenchyme transition
ESCRT	endosomal sorting complexes required for transport
EVs	extracellular vesicles
FDCs	Follicular dendritic cells
GR	glucocorticoid receptor
GTPases	guanosine triphosphase
HCC	hepatocellular carcinoma
HDAC6	histone deacetylase 6
HL	Hodgkin’s lymphoma
hnRNPA2B1	Heterogeneous Nuclear Ribonucleoproteins A2/B1
HSCs	hematopoietic stem cells
HUVECs	human vascular endothelial cells
ICAM-1	intercellular adhesion molecule-1
IL	interleukin
ILV	intraluminal vesicles
IM	imatinib
IVs	intracellular vesicles
LAM	lymphoma-associated macrophages
lncRNAs	long non-coding RNAs
LSCs	leukemic stem cells
MAPK	MAP kinases
MDR	multiple-DR
MGUS	Monoclonal Gammopathy of Indeterminate Significance
miRNA	micro-RNA
MM	Multiple myeloma
MMP	matrix metalloproteinase
MSC	mesenchymal stem/stromal cells
mTOR	mechanistic target of rapamycin
mRNA	messenger RNA
MSCs	mesenchymal stem cells
MVBs	multivesicular bodies
NF-kB	nuclear factor kappa-light-chain-enhancer of activated B cells
NHL	non-Hodgkin’s lymphoma
NK	natural killer
OPG	degrading osteoprotegerin
OXPHOS	oxidative phosphorylation
PARP	ADP-ribose polymerase
PCs	plasmacells
P-gp	p-glycoprotein
Ph	Philadelphia chromosome
PI	proteasome inhibitor
PI3K	PhosphatidylInositol 3-Kinase
PKM2	Pyruvate Kinase M2
PTEN	Phosphatase and tensin homolog
RS	Reed Sternberg cell
SF	soluble factors
SPHK2	Sphingosine-1-phosphate, sphingosine kinase 2
SMART-exos	Synthetic Multivalent Antibodies ReTargeted Exosomes
SNARE	the soluble *N*-ethylmaleimide-sensitive factor attachment protein receptor
TAFs	Tumor-associated fibroblasts
TAMs	tumor-associated macrophages
TASCs	Tumor-associated stromal cells
TGFβ1	Transforming growth factor beta 1
Th	helper T cells
TFH	follicular helper T cells
TKIs	tyrosine kinase inhibitors
TME	Tumor microenvironment
Treg	regulatory T cells
VEGF	Vascular-Endothelial Growth Factor
VCAM-1	vascular cell adhesion molecule-1
VTA1	vesicle trafficking
